# Rare Variants in Purinergic P2X Receptor Genes *(P2RX4, P2RX5, P2RX7)* in Individuals With Autism Spectrum Disorder: An Exploratory Study

**DOI:** 10.1155/humu/5522396

**Published:** 2026-07-18

**Authors:** Gül Ünsel-Bolat, Hilmi Bolat

**Affiliations:** ^1^ Department of Child and Adolescent Psychiatry, Faculty of Medicine, Balıkesir University, Balıkesir, Türkiye, balikesir.edu.tr; ^2^ Department of Medical Genetics, Faculty of Medicine, Balıkesir University, Balıkesir, Türkiye, balikesir.edu.tr

**Keywords:** autism spectrum disorder, genetic variants, maternal immune activation, neuroinflammation, P2X receptors, purinergic signaling, whole-exome sequencing

## Abstract

**Background:**

Purinergic P2X receptors (*P2RX*) play key roles in neuroinflammatory processes through ATP‐gated ion channel signaling. Dysregulation of *P2RX* receptor activity has been implicated in maternal immune activation, mitochondrial dysfunction, oxidative stress, and synaptic abnormalities associated with autism spectrum disorder (ASD). Despite increasing mechanistic evidence, the contribution of *P2RX* gene variants to ASD remains poorly understood.

**Methods:**

We retrospectively evaluated whole‐exome sequencing (WES) data from individuals diagnosed with ASD according to DSM‐5 criteria. Variants in the *P2RX1–P2RX7* genes were identified. Clinical, genetic, and in silico data were reviewed, and variants were classified according to ACMG/AMP guidelines.

**Results:**

Eleven individuals were identified with heterozygous *P2RX* variants, most frequently in *P2RX7* (54.5%), followed by *P2RX5* (27.3%) and *P2RX4* (18.2%). Core clinical features across all genes included ASD, intellectual disability (ID), and epilepsy. *P2RX4* variants included an in‐frame deletion (p.Ile322del) and a truncating variant (p.Tyr366LeufsTer7), both observed in individuals with ASD and ID. *P2RX5* variants comprised rare missense substitutions with variable in silico pathogenicity. Several *P2RX7* variants, most notably the canonical splice‐site variant c.614 + 1G > A fulfilling ACMG/AMP PVS1 and PM2 criteria, were predicted to alter receptor function. Overall, in silico analyses indicated moderate to high predicted functional impact; however, most variants lacked segregation and functional validation data.

**Conclusion:**

Our findings provide preliminary, exploratory observations suggesting that rare *P2RX* variants may represent biologically plausible candidates relevant to ASD‐related pathways; however, no conclusions regarding disease association or causality can be drawn. These findings should be considered hypothesis‐generating and require validation through larger controlled genetic studies and functional investigations.

## 1. Introduction

Purinergic P2X receptors (P2RX) are a family of ATP‐gated ion channels that mediate fast excitatory neurotransmission and regulate numerous physiological and pathological processes in the central nervous system (CNS). Among these, P2X4 and P2X7 receptors have received considerable attention due to their central roles in neuroinflammation and neurodegeneration. Activation of P2X4 receptors in microglia triggers inflammatory cascades through cytokine release [1, 2] and promotes apoptosis, lesion expansion, and worsened neurological outcomes [3, 4]. Similarly, stimulation of the P2X7 receptor contributes to blood–brain barrier disruption [5], astrogliosis [6], apoptosis [7], and exacerbated lesion progression [8], underscoring its pivotal role in neuroinflammatory processes [9].

P2X7R is a key mediator of IL‐1*β* release following immune activation, linking purinergic signaling to cytokine‐driven behavioral responses and neuroimmune dysregulation [10]. Recent genetic studies have further implicated P2RX7 variants in psychiatric disease vulnerability [11], indicating that altered P2X7 signaling may influence neuroimmune and neurotransmission pathways relevant to neuropsychiatric disease.

Accumulating evidence also implicates P2X7 receptor upregulation in autism spectrum disorder (ASD). Enhanced ATP‐mediated P2X7 activation has been proposed to participate in maternal immune activation, mitochondrial dysfunction, oxidative stress, and chronic neuroinflammation, all biological processes relevant to ASD pathophysiology [12]. Mechanistically, P2X7R activation allows sodium, calcium, and potassium flux across the membrane, whereas prolonged stimulation generates large pores that facilitate apoptotic and inflammatory signaling [12]. Preclinical work has shown that pharmacological inhibition or genetic blockade of P2X7 can ameliorate ASD‐like features in rodent models [13, 14], highlighting purinergic receptors as promising therapeutic targets.

Despite these advances, the clinical relevance of genetic variations in *P2RX* genes in ASD remains poorly understood. Most mechanistic studies have focused on receptor function or pharmacological modulation, whereas human genetic contributions have remained limited and incompletely characterized. Given the established involvement of purinergic receptors in neuroinflammatory and neurodevelopmental pathways, investigating genetic variations in ASD may provide additional insight into biologically relevant mechanisms underlying neurodevelopmental phenotypes.

In this study, we performed an exploratory analysis of whole‐exome sequencing (WES) data to investigate the possible relevance of rare *P2RX* gene variants identified in individuals with ASD. Given the descriptive design of the study, our findings were intended to generate hypotheses and identify biologically plausible candidate pathways for future investigation rather than establish disease association or causality.

## 2. Materials and Methods

Ethical approval for this study was obtained from the Balıkesir University Ethics Committee on November 4, 2025, under decision number 2025/9‐12.

### 2.1. Study Population and Case Selection

Clinical and genetic data from individuals referred to the Medical Genetics and Child and Adolescent Psychiatry clinics of Balıkesir University and diagnosed with ASD were retrospectively reviewed. A total of 110 individuals with ASD who underwent WES as part of their clinical diagnostic evaluation were initially screened.

All cases were included if they met the criteria for ASD in the Diagnostic and Statistical Manual of Mental Disorders, Fifth Edition (DSM‐5). We reviewed their medical records to collect their clinical information.

This was a retrospective cross‐sectional study. Inclusion criteria were (i) confirmed ASD diagnosis according to DSM‐5 criteria, (ii) normal molecular karyotyping results, (iii) normal Fragile X testing results, (iv) absence of previously established pathogenic or likely pathogenic variants associated with neurodevelopmental disorders, and (v) availability of WES data and sufficient clinical information. Individuals in whom a genetic diagnosis explaining ASD or neurodevelopmental findings had already been established were excluded from the study. Therefore, the present analysis focused specifically on individuals without an established genetic diagnosis.

WES datasets were screened using custom gene sets including *P2RX1–P2RX7* genes involved in purinergic signaling pathways. Following filtering and prioritization, individuals carrying heterozygous *P2RX4, P2RX5,* or *P2RX7* variants were included in the descriptive analysis.

No control cohort enrichment or burden analysis was performed because the aim of this study was the descriptive characterization of rare *P2RX* variants identified within a clinically referred ASD cohort.

### 2.2. Genetic Testing and Analysis

Written informed consent was obtained from all participants prior to blood sample collection. Genomic DNA was extracted from 200 *μ*L peripheral blood samples using the HiPurA prefilled clinical multipurpose nucleic acid purification kit and the HIMEDIA InstaN Mag‐96 automated extraction system according to the manufacturer′s instructions. DNA concentration and quality were assessed using a Qubit fluorometer (Thermo Fisher Scientific, United States).

WES libraries were prepared using the Roche KAPA HyperExome 96 reaction kit according to the manufacturer′s protocol and sequenced on the MGI DNBSEQ‐G400 platform. Sequencing achieved an average on‐target depth of 80–100x with an average target coverage of 97.17%, and mean read depth exceeding ×20 across targeted regions. Exon–intron junction boundaries (±10 bp) were included in the analysis to facilitate identification of potential splice‐site variants.

Variant classification and analysis were performed using the SEQ Platform (Genomize Inc.). FASTQ files were uploaded to the SEQ Platform and processed by aligning sequencing reads to the human reference genome hg38/GRCh38 using the Burrows–Wheeler Aligner (BWA) [15]. Aligned reads were subsequently used for variant calling with FreeBayes [16]. PCR duplicate removal and local indel realignment were performed using proprietary algorithms implemented within the Genomize SEQ Platform. Identified variants were annotated using Variant Effect Predictor (VEP) V102 [17].

Quality control assessment included evaluation of sequencing depth, read support, mapping quality, variant allele fraction, and overall variant quality metrics generated by the analytical pipeline. Variants supported by fewer than five reads or exhibiting a variant allele fraction below 20% were excluded from downstream analyses. Additional variants with insufficient coverage or poor‐quality metrics were also removed prior to interpretation.

ACMG/AMP variant classification was performed using Genomize′s proprietary interpretation algorithm based on the recommendations of Richards et al. [18]. Additional evidence used for variant interpretation included ClinVar annotations, in silico prediction scores available within the SEQ Platform, and population frequency data. Variant annotation and filtering were performed within the same analytical environment to ensure methodological consistency. Human Phenotype Ontology (HPO)–based filtering was applied for phenotype‐driven prioritization, and gene selection was guided using Online Mendelian Inheritance in Man (OMIM). Rare protein‐altering variants, including missense, nonsense, splice‐site, frameshift, and in‐frame variants, were retained for further evaluation. Variants with a minor allele frequency (MAF) > 0.5% in population databases were excluded from further analysis. Candidate variants within genes of interest, including *P2RX* family genes, were subsequently prioritized according to predicted functional consequence, phenotypic relevance, and compatibility with the observed phenotype. Pathogenicity assessment was performed using multiple in silico prediction tools, including MutationTaster, Combined Annotation Dependent Depletion (CADD), DANN, and AlphaMissense. Variants of interest were additionally reviewed manually to minimize potential analytical artifacts. Variant interpretation and classification followed the American College of Medical Genetics and Genomics/Association for Molecular Pathology (ACMG/AMP) guidelines and variants were categorized as pathogenic, likely pathogenic, variant of uncertain significance (VUS), likely benign, or benign. A detailed summary of ACMG/AMP evidence codes and final classifications for all reported variants is provided in Table S1.

Sanger sequencing confirmation was not performed for variants identified in this retrospective study. Variants were interpreted based on sequencing quality metrics, annotation resources, computational prediction tools, and ACMG/AMP classification criteria.

## 3. Results

A total of 11 individuals were identified with heterozygous variants in *P2RX* genes (*P2RX4, P2RX5,* and *P2RX7*) using WES data. All variants were classified according to ACMG/AMP criteria. The majority of individuals presented with ASD, either isolated or in combination with intellectual disability (ID) and/or epilepsy. Detailed clinical and genetic findings are summarized in Table 1.

**Table 1:** Genetic and clinical findings of P2X receptor gene variants.

Case no.	Age	Gender	Gene transcript	Variation	Location	Variant type	gnomAD frequency (total)	ACMG pathogenicity criteria	ClinVar	Conservation scores phyloP100	CADD	AlphaMissense	DANN	Associated phenotype
1	6	M	P2RX4 (NM_002560.3)	c.964_966del p.(Ile322del)	Exon 9	In frame	0.0001301	VUS (PM4, PM2)	Not reported	7.599	—	—	—	ASD
2	8	M	P2RX4 (NM_002560.3)	c.964_966del p.(Ile322del)	Exon 9	In frame	0.0001301	VUS (PM4, PM2)	Not reported	7.599	—	—	—	ASD, ID
3	6	F	P2RX4 (NM_002560.3)	c.1095_1096del p.(Tyr366LeufsTer7)	Exon 11	Frameshift	0.000003718	VUS (PM2)	Not reported	3.847	—	—	—	ASD, ID
4	4	M	P2RX5 (NM_002561.4)	c.556G > A p.(Glu186Lys)	Exon 6	Missense	0.0002868	VUS (PM2)	Not reported	4.932	Pathogenic 26.5	Benign 0.1214	Uncertain 0.9984	ASD
5	4	M	P2RX5 (NM_002561.4)	c.729C > A p.(Ser243Arg)	Exon 7	Missense	No variant	VUS (PM2, BP4)	Not reported	‐0.251	Benign 8.1079	Uncertain 0.5129	Benign 0.9387	ASD, Epilepsy
6	7	M	P2RX7 (NM_002562.6)	c.1733G > A p.(Arg578Gln)	Exon 13	Missense	0.005183	Benign (PM2, BS2, BP6)	Reported (likely benign)	7.119	Pathogenic 28.2999	Benign 0.2228	Pathogenic 0.9995	ASD, Epilepsy
7	5	M	P2RX7 (NM_002562.6)	c.462_463delGTinsAC p.(Tyr155His)	Exon 5	Missense	No variant	VUS (PM2)	Not reported	6.547	—	—	—	ASD, Epilepsy
8	7	F	P2RX7 (NM_002562.6)	c.462_463delGTinsAC p.(Tyr155His)	Exon 5	Missense	No variant	VUS (PM2)	Not reported	6.547	—	—	—	ASD
9	5	M	P2RX7 (NM_002562.6)	c.462_463delGTinsAC p.(Tyr155His)	Exon 5	Missense	No variant	VUS (PM2)	Not reported	6.547	—	—	—	ASD, Epilepsy
10	8	M	P2RX7 (NM_002562.6)	c.614 + 1G > A	Intron 6	Splice‐site	0.00009555	LP (PVS1, PM2)	Not reported	8.084	Pathogenic 34	—	Uncertain 0.9956	ASD, Epilepsy
11	6	M	P2RX7 (NM_002562.6)	c.886G > A p.(Ala296Thr)	Exon 9	Missense	0.00006030	VUS (PM2)	Not reported	5.147	Pathogenic 26.6	Uncertain 0.3954	Pathogenic 0.9993	ASD

Abbreviations: ASD, autism spectrum disorder; F, female; ID, intellectual disability; M, male; VUS, variant unknown significance.

Across all *P2RX* genes, *P2RX7* variants represented the largest subset (54.5%), followed by *P2RX5* (27.3%) and *P2RX4* (18.2%). Phenotypic overlap was notable across these genes, with ASD and ID as the most consistent clinical features, and epilepsy identified in 45.5% of cases. In silico predictions indicated moderate to high functional impact for several variants; however, most variants remained classified as VUS and lacked segregation or experimental functional validation.

### 3.1. *P2RX4* Gene Variants

Three patients carried variants in *P2RX4* (NM_002560.3). Two unrelated males (Cases 1 and 2) shared an in‐frame deletion c.964_966del (p.Ile322del) in Exon 9, reported at low population frequency (gnomAD: 0.0001301). Both exhibited ASD, and one also had ID. A third patient (Case 3) harbored a frameshift variant c.1095_1096del (p.Tyr366LeufsTer7) predicted to generate premature protein truncation and was identified in a female with ASD and ID (Table 1).

### 3.2. *P2RX5* Gene Variants

Two patients (Cases 4 and 5) carried heterozygous variants in *P2RX5* (NM_002561.4). The variants c.556G > A (p.Glu186Lys) and c.729C > A (p.Ser243Arg) were identified in individuals presenting with ASD, one of whom also had epilepsy. These variants demonstrated variable and sometimes discordant predictions across multiple in silico algorithms, including CADD, DANN, and AlphaMissense. Both variants remained classified as VUS according to ACMG criteria (Table 1).

### 3.3. *P2RX7* Gene Variants

Six individuals carried variants in *P2RX7* (NM_002562.6). A canonical splice donor variant, c.614 + 1G > A, affecting the invariant +1 position, was identified in one individual with ASD and epilepsy. This variant fulfilled ACMG/AMP PVS1 and PM2 criteria and was classified as likely pathogenic.

Additional variants included c.1733G > A (p.Arg578Gln), c.462_463delGTinsAC (p.Tyr155His), observed in three unrelated individuals, and c.886G > A (p.Ala296Thr). These variants demonstrated heterogeneous in silico predictions and were classified as benign or VUS according to ACMG criteria. Although several variants were absent or extremely rare in population databases, interpretation remained limited by the absence of segregation analysis and functional validation (Table 1).

## 4. Discussion

Our findings provide preliminary and exploratory observations regarding rare variants identified in *P2RX* genes in individuals with ASD. Given the descriptive design of the study and the absence of a control cohort, these findings should not be interpreted as evidence of disease association or causality. Rather, they identify biologically plausible candidate pathways that warrant further investigation. The identification of rare variants in *P2RX4* and *P2RX7* within our cohort highlights purinergic signaling pathways as biologically plausible candidates for further investigation in ASD. Further functional and segregation studies are warranted to clarify their pathogenic significance and mechanistic consequences.

### 4.1. *P2RX4* Variants and Neurodevelopmental Pathways

We identified rare *P2RX4* variants in three individuals with ASD, two of whom also presented with ID. These human genetic findings are consistent with, rather than confirm, experimental evidence demonstrating that *P2X4* receptors are actively involved in neurodevelopmental processes. In animal models, *P2X4* receptors were associated with abnormalities in sensory processing, impaired prepulse inhibition, enhanced tactile sensitivity, and reduced sociocommunicative behaviors, including diminished social interaction and decreased maternal separation–induced ultrasonic vocalizations [19]. These behavioral phenotypes show similarities to core features observed in ASD.

Additionally, *P2RX4* knockout mice were reported to display region‐specific alterations in ionotropic glutamate receptor subunit composition, suggesting that *P2RX4* perturbation affects excitatory synaptic organization. Furthermore, *P2RX4* is known to modulate major ionotropic targets, including NMDA glutamate receptors, which are central to cognitive and emotional processing and have been implicated in ASD. These mechanistic links support biological plausibility but do not establish direct evidence of pathogenicity in humans [20–22].

### 4.2. *P2RX7* Variants and Neurodevelopmental Pathways

Recent research has highlighted P2X7 receptor upregulation in ASD and its involvement in pathological processes such as maternal immune activation, mitochondrial dysfunction, oxidative stress, and chronic neuroinflammation. Experimental evidence suggests that *P2RX7* signaling may influence neuroimmune responses, and receptor alterations may participate in biological pathways relevant to ASD pathophysiology [12].

Among *P2RX7* findings, we identified several rare variants with heterogeneous ACMG classifications and in silico predictions. Notably, the c.462_463delGTinsAC (p.Tyr155His) variant was observed in three unrelated individuals, making it the most recurrent *P2RX7* variant within our cohort. Although recurrence within this small dataset may be noteworthy, interpretation remains limited because segregation data and functional evidence are unavailable.

We also draw particular attention to the *P2RX7* (NM_002562.6) c.614 + 1G > A variant identified in our study. This variant disrupts the canonical +1 splice donor site, a highly conserved nucleotide essential for accurate pre‐mRNA splicing. Evolutionary conservation metrics (phyloP100way: 8.084; PhastCons100way: 1.0) further highlight the strong evolutionary constraint at this position. Loss of the invariant splice donor site is predicted to cause aberrant splicing, potentially resulting in premature termination, frameshifted transcripts, or nonsense‐mediated mRNA decay, ultimately leading to loss of P2X7 receptor function. According to ACMG/AMP variant interpretation guidelines, this variant meets PVS1 and PM2 criteria, supporting its classification as likely pathogenic. However, segregation analyses and experimental validation were unavailable, and the relationship between *P2RX7* loss‐of‐function and ASD has not yet been definitively established.

### 4.3. Therapeutic Implications of Purinergic Antagonism

The role of purinergic signaling in ASD has prompted interest in P2X/P2Y receptor antagonists as potential therapeutic agents. Suramin, an established antipurinergic and anti‐inflammatory compound, has shown promising preclinical efficacy. In animal maternal immune activation models, a single 20 mg/kg dose of suramin restored social behavior, normalized novelty preference, and corrected metabolic disturbances [13]. Similar improvements have been observed in Fragile X knockout mice [23]. These results led to human trials. In an initial pilot study (*n* = 10), a single 20 mg/kg intravenous dose of suramin was associated with improvements in language, social communication, and reductions in repetitive behaviors [24]. A larger randomized, double‐blind, placebo‐controlled study (*N* = 52) evaluated monthly infusions over 14 weeks [25]. Although the primary ABC‐Core outcome did not reach statistical significance, the 10 mg/kg arm demonstrated meaningful clinical benefit on the CGI‐I scale, with favorable responses particularly in younger children and those with less severe baseline symptoms. In addition, authors reported that suramin was well‐tolerated during the study.

Importantly, no clinical studies have specifically evaluated antipurinergic compounds in individuals carrying *P2RX* variants. Therefore, any genotype‐specific therapeutic implications remain speculative and require dedicated investigation. Stratified clinical studies will be necessary to determine whether specific genetic backgrounds influence treatment response.

### 4.4. Study Limitations

Several limitations should be considered when interpreting the present findings. First, the relatively small sample size limits statistical inference and prevents causal conclusions. Second, no control cohort or case–control burden analysis was included; therefore, the identified variants cannot be interpreted as evidence of ASD risk or genetic association. Third, segregation analyses were unavailable for most individuals, preventing assessment of inheritance patterns, penetrance, and familial contribution. Fourth, experimental functional validation and orthogonal confirmation methods, such as Sanger sequencing, were not performed. Although the identified variants met predefined quality‐control criteria, the absence of independent validation may reduce confidence in variant identification and interpretation. Future studies should incorporate orthogonal validation approaches whenever feasible to further strengthen the reliability of genetic findings. Finally, interpretation of rare variants remains challenging in ASD because of substantial genetic heterogeneity and the possibility that some identified variants may represent low‐effect susceptibility alleles rather than high‐impact pathogenic variants. Accordingly, these findings should be considered exploratory and hypothesis‐generating observations.

In summary, our findings identify rare *P2RX* variants in a clinically characterized ASD cohort and support the biological plausibility of purinergic signaling pathways as candidates for future investigation. Given the limited sample size, absence of a control cohort, and lack of functional validation, these findings should be considered exploratory and hypothesis‐generating. Larger controlled genetic studies and experimental investigations will be necessary to clarify the role of *P2RX* variants in ASD and their potential relevance for precision medicine approaches.

## Funding

No funding was received for this manuscript.

## Conflicts of Interest

The authors declare no conflicts of interest.

## Supporting information


**Supporting Information** Additional supporting information can be found online in the Supporting Information section. Table S1: ACMG/AMP classification of identified P2RX variants. The table summarizes all identified P2RX gene variants and their classification according to the American College of Medical Genetics and Genomics/Association for Molecular Pathology (ACMG/AMP) guidelines. Evidence codes and the final pathogenicity classification assigned to each variant are provided.

## Data Availability

The data that support the findings of this study are available from the corresponding author upon reasonable request.
