# Supplementary material for: Rare Variants in Purinergic P2X Receptor Genes (P2RX4, P2RX5, P2RX7) in Individuals With Autism Spectrum Disorder: An Exploratory Study
Source: Hum Mutat. 2026 Jul 18;2026:5522396. doi: 10.1155/humu/5522396 (PMC13379949; doi:10.1155/humu/5522396)
Supplement: Supplementary file 1 — Supporting Information Additional supporting information can be found online in the Supporting Information section. Table S1: ACMG/AMP classification of identified P2RX variants. The table summarizes all identified P2RX gene variants and their classification according to the American College of Medical Genetics and Genomics/Association for Molecular Pathology (ACMG/AMP) guidelines. Evidence codes and the final pathogenicity classification assigned to each variant are provided. [file HUMU-2026-5522396-s001.docx]

# Supplementary Table S1. ACMG/AMP classification of identified *P2RX* variants

| Gene | Variant (HGVS) | Protein Change | ACMG Evidence Codes | Final Classification |
| --- | --- | --- | --- | --- |
| *P2RX4* | c.964_966del | p.(Ile322del) | PM4, PM2 | VUS |
| *P2RX4* | c.1095_1096del | p.(Tyr366LeufsTer7) | PM2 | VUS |
| *P2RX5* | c.556G>A | p.(Glu186Lys) | PM2 | VUS |
| *P2RX5* | c.729C>A | p.(Ser243Arg) | PM2, BP4 | VUS |
| *P2RX7* | c.1733G>A | p.(Arg578Gln) | PM2, BS2, BP6 | Benign |
| *P2RX7* | c.462_463delGTinsAC | p.(Tyr155His) | PM2 | VUS |
| *P2RX7* | c.614+1G>A | — | PVS1, PM2 | Likely Pathogenic |
| *P2RX7* | c.886G>A | p.(Ala296Thr) | PM2 | VUS |
